# Income inequality, life expectancy and cause-specific mortality in 43 European countries, 1987–2008: a fixed effects study

**DOI:** 10.1007/s10654-015-0066-x

**Published:** 2015-07-16

**Authors:** Yannan Hu, Frank J. van Lenthe, Johan P. Mackenbach

**Affiliations:** Department of Public Health, Erasmus University Medical Centre, PO Box 2040, 3000 CA Rotterdam, The Netherlands

**Keywords:** Europe, Mortality, Life expectancy, Causes of death, Income inequality, Fixed effects models

## Abstract

Whether income inequality is related to population health is still open to debate. We aimed to critically assess the relationship between income inequality and mortality in 43 European countries using comparable data between 1987 and 2008, controlling for time-invariant and time-variant country-level confounding factors. Annual data on income inequality, expressed as Gini index based on net household income, were extracted from the Standardizing the World Income Inequality Database. Data on life expectancy at birth and age-standardized mortality by cause of death were obtained from the Human Lifetable Database and the World Health Organization European Health for All Database. Data on infant mortality were obtained from the United Nations World Population Prospects Database. The relationships between income inequality and mortality indicators were studied using country fixed effects models, adjusted for time trends and country characteristics. Significant associations between income inequality and many mortality indicators were found in pooled cross-sectional regressions, indicating higher mortality in countries with larger income inequalities. Once the country fixed effects were added, all associations between income inequality and mortality indicators became insignificant, except for mortality from external causes and homicide among men, and cancers among women. The significant results for homicide and cancers disappeared after further adjustment for indicators of democracy, education, transition to national independence, armed conflicts, and economic freedom. Cross-sectional associations between income inequality and mortality seem to reflect the confounding effects of other country characteristics. In a European context, national levels of income inequality do not have an independent effect on mortality.

**Electronic supplementary material:**

The online version of this article (doi:10.1007/s10654-015-0066-x) contains supplementary material, which is available to authorized users.

## Introduction

Whether income inequality harms population health is still open to debate. Since Wilkinson [1] postulated the hypothesis that income inequality was not simply a summary of the balance of income between the rich and poor, but is a health risk in its own right [2], a wide array of studies, including multilevel studies within countries and cross-country ecological studies examined the link between income distribution and population health [3, 4]. However, no agreement has yet been reached because of discrepancies between the results of different studies.

International comparative studies linking income inequality to mortality suffer from limited comparability of the income inequality measures between countries and over time [5–7]. The Luxembourg Income Study (LIS) [8], regarded as the “gold standard”, is the first choice for many studies [1, 5, 9, 10] because of its high quality and comparability. It covers, however, only a limited set of country-year observations, which may be the reason why many studies using this database performed a cross-sectional analysis. The Deininger and Squire database (1996) is often chosen as an alternative source [6, 11, 12] and provides more observations, but at a substantial loss of comparability. The World Income Inequality Database (WIID) covers the most comprehensive set of income inequality statistics. It incorporates several data sources, and enables researchers to maximize comparability by choosing data based on the criteria of comparability, but potentially leads to a risk of not piecing together the information in a meaningful way [13, 14]. The more recently developed Standardizing the World Income Inequality Database (SWIID) maximizes comparability for the broadest available set of country-year observations, and as such is better suited than other income inequality datasets for cross-country comparative research [14].

With some exceptions [9, 12], cross-sectional studies found significantly worse population health at higher levels of income inequality [1, 15–17]. However, these associations sometimes diminished after adjustment for observed country characteristics [3, 4, 6, 11, 18], suggesting there is a substantial risk of confounding. Fixed effect models, which require longitudinal data, are able to adjust for unobservable time-invariant confounding variables, by linking changes in income inequality to changes in health. Studies using fixed effects models to study the effect of income inequality on population health often reported insignificant results [6, 7, 11, 19–21]. However, these studies pooled men and women together [6, 7, 10, 11, 19, 22, 23], used relatively old data [6, 11, 19], restricted the outcome to infant mortality [21], or ignored some potential time-variant confounders [6, 10, 19, 20, 23, 24]. Only few studies investigated disease-specific outcomes, which would help to interpret findings on the basis of existing knowledge on determinants of population health and could point towards potential pathways through which income inequality may harm population health [7, 11, 19]. Studies specifically assessing the association between income inequality and mortality in a European context, which would be important for policy makers in Europe, are also limited in number [25, 26].

Using the SWIID data, we therefore aimed to refine and extend previous studies by critically investigating the relationships between income inequality and a set of disease-specific mortality indicators by gender in fixed effects models for 43 European countries over the period 1987–2008 [21].

## Data and methods

### Data

For income inequality, we made use of a new dataset called Standardizing the World Income Inequality Database (SWIID). Using the Gini index as measure, SWIID took version 2.0c of the WIID [27] as the starting-point and standardized it based on the inequality observations from the LIS [8]. Standardizing procedures were applied to account for differences in (a) population coverage (e.g. whether data cover all or nearly all of a country’s population), (b) income reference units (e.g. household per capita, household adult equivalent, or household without adjustment of number of people), and (c) the definition of income (e.g. net income, gross income, expenditures or unidentified income). Finally, missing observations were imputed based on proximate years using a custom multiple-imputation algorithm [14]. In this study, we extracted information on the Gini index based on net household income (post-tax post-transfer) from SWIID version 4.0 covering 43 European countries with 879 country-year observations.

Data on life expectancy at birth and age-standardized mortality by cause of death at all ages (further referred to as “mortality indicators”) were extracted from the Human Lifetable Database (www.lifetable.de) and the World Health Organization European Health for All Database (http://data.euro.who.int/hfadb/). Data on infant mortality, measured as infant deaths per 1000 live births, were obtained from the United Nations World Population Prospects database (http://esa.un.org/wpp/Excel-Data/mortality.htm). All mortality rates are log-transformed for normalization. ICD-code numbers are reported in a previous paper [28].

A key variable potentially confounding the relationship between income inequality and mortality, and for which the majority of existing studies controlled, is national income, which was measured by gross domestic product (GDP) per head of population (in $1000s, extracted from a dataset compiled by Maddison http://www.ggdc.net/MADDISON/oriindex.htm). Besides GDP, a number of other potential confounders were added where appropriate, including indicators of democracy (the Policy2 index ranging from “strongly democratic (+10)” to “strongly autocratic (−10)”, extracted from the Quality of Government dataset, http://www.qog.pol.gu.se/data/), average years of schooling (extracted from the Barro-Lee Educational Attainment dataset, http://www.barrolee.com/, made into annual data by linear interpolation), transition to national independence (0 for no transition, 1 for the year of independence and the three subsequent years), armed conflict (ranging from 0 for “no conflict” to 3 for “more than 1000 battle deaths per year”, constructed using data on conflict location extracted from the Quality of Government dataset, http://www.qog.pol.gu.se/data/), and economic freedom (ranging from 0 to 10, extracted from the Economic Freedom of the World 2011 dataset and imputed some missing data for earlier periods using the 2002 dataset update, http://www.freetheworld.com/). Indicators of democracy, education and economic freedom were chosen because they represent changes in the underlying political, social and financial conditions prevailing in each country. Indicators of transition to national independence and armed conflicts were chosen to account for the disruption of governance structures and political changes in Central and Eastern Europe, and some Mediterranean and Western Balkan countries during the study-period. All these variables have been documented to affect population health in Europe [28] and potentially correlate with but may not be seen as causally resulting from income inequality [29–31].

### Analytical approach

To align with previous studies, we first explored the pooled cross-sectional relation between income inequality and mortality. We adjusted analyses for year dummies and GDP per capita, and subsequently included more confounding variables. Robust standard errors were used to account for heteroskedasticity [32].

The model can be written as:

$$healthoutcome_{ij} = \alpha + \beta_{1} Gini_{ij} + \beta_{2} \ln gdp_{ij} + T_{j} + \gamma C_{ij}$$

where health outcome_ij_ is the life expectancy or logarithmic form of the age-adjusted mortality rates for country i in year j; α is a constant; Gini_ij_ represents the Gini index; lngdp_ij_ is the logarithmic form of GDP per head, accounting for the potential non-linear relationship between national income and health; T_j_ is a vector of year dummies controlling the shared time trend in mortality during the study period; C_ij_ represents other potential time-variant confounders including indicators of democracy, years of schooling, independence, armed conflict and economic freedom, which were added subsequently.

As the next step, we applied fixed effects models, which allowed to control for unobserved time-invariant country heterogeneity, such as cultural, social, historical, geographic and other conditions that remained relatively constant within the study period. The results of the fixed effects models can be interpreted as the relationship between annual changes in income inequality and annual changes in health outcomes. Clustered sandwich estimators were used to allow for within-country correlation between error terms [32].

The model can be written as:

$$healthoutcome_{ij} = \alpha + \beta_{1} Gini_{ij} + \beta_{2} \ln gdp_{ij} + X_{i} + T_{j} + \gamma C_{ij}$$

where X_i_ is a vector of country fixed effects.

In the online supplementary material, supplementary analyses checking robustness of the results are reported, which include (a) models allowing a different linear time trend and different effects of the country characteristics for the former Soviet countries (Supplementary Table 2); (b) models allowing country-specific linear time trends by including the interaction terms between country dummies and year (Supplementary Table 2); (c) analyses restricted to high-income countries within Europe (Supplementary Table 3); (d) analyses using the Gini index based on gross instead of net household income (Supplementary Table 4); (e) analyses sequentially replacing contemporaneous Gini indexes by indexes up to 10 years before the mortality outcomes (“lagged terms”) (Supplementary Table 5); (f) analyses using the Gini index from the LIS instead of the SWIID (Supplementary Table 6).

All regression analyses were performed in Stata 13.1.

## Results

Table 1 reports descriptive information on the Gini index, life expectancy and infant mortality in the period between 1987 and 2008 for each country (other outcomes are described in the Supplementary Table 1). In some countries, information on the Gini index only became available in more recent periods (e.g. Iceland, Cyprus, Albania, Bosnia Hercegovina, Malta, Montenegro and Serbia). The Gini index ranges from 15.77 (Slovakia in 1989) to 47.94 (Azerbaijan in 1997). The mean Gini index over the whole period was generally lower in the Nordic region, and higher in Britain and Ireland, and in countries of the former Soviet Union. Higher standard deviations of the Gini index reflect higher within-country variations over time, which particularly occurred in countries of the former Soviet Union and the Western Balkans. The mean life expectancy was lowest in former Soviet countries, followed by countries of the Western Balkans and Central and Eastern Europe. They were relatively similar to each other in the other four European regions. Infant mortality was highest in former Soviet countries, followed by countries of the Western Balkans and Central and Eastern Europe. Life expectancy of women was higher than life expectancy of men in all countries.

**Table 1:** Descriptive information on income inequality (GINI), life expectancy and infant mortality between 1987 and 2008 in 43 European countries

Country	Gini index^a^			Life expectancy at birth				All-cause mortality				Infant mortality		
	Years	N	Mean (SD)	Years	N	Mean (men)	Mean (women)	Years	N	Mean (men)	Mean (women)	Years	N	Mean
Nordic countries														
Denmark	1987–2008	22	23.37 (0.28)	1987–2006	20	73.81	78.90	1987–2006	20	993.57	656.05	1987–2008	22	5.55
Finland	1987–2008	22	23.25 (0.46)	1987–2008	22	73.55	80.98	1987–2008	22	996.25	552.38	1987–2008	22	4.15
Iceland	1992–2008	17	24.77 (0.60)	1987–2008	22	77.47	81.76	1987–2008	22	740.79	513.66	1987–2008	22	3.77
Norway	1987–2008	22	23.59 (0.20)	1987–2008	22	75.66	81.36	1987–2008	22	865.36	530.08	1987–2008	22	4.82
Sweden	1987–2008	22	23.14 (0.29)	1987–2008	22	76.82	81.89	1987–2008	22	795.45	506.83	1987–2008	22	4.15
Britain and Ireland														
Ireland	1987–2008	22	31.98 (0.32)	1987–2008	22	74.05	79.40	1987–2008	22	1005.51	640.92	1987–2008	22	6.13
United Kingdom	1987–2008	22	33.93 (0.29)	1987–2008	22	74.93	79.95	1987–2008	22	912.91	599.70	1987–2008	22	6.30
Continental Europe														
Austria	1987–2008	22	26.93 (0.45)	1987–2008	22	74.62	80.81	1987–2008	22	919.51	558.54	1987–2008	22	5.82
Belgium	1987–2008	22	25.00 (0.41)	1987–2005	15	73.68	80.31	1987–2005	15	991.75	573.11	1987–2008	22	5.97
Luxembourg	1987–2008	22	25.60 (0.35)	1987–2008	22	74.20	80.76	1987–2008	22	957.18	560.91	1987–2008	22	5.66
Germany (UnitedNations)	1987–2008	22	27.15 (0.20)	1987–2008	22	74.44	80.50	1990–2006	17	933.29	565.48	1987–2008	22	5.21
Switzerland	1987–2008	22	29.34 (0.44)	1987–2007	21	76.40	82.55	1987–2007	21	794.44	469.89	1987–2008	22	5.08
Netherlands	1987–2008	22	25.47 (0.28)	1987–2008	22	75.49	80.95	1987–2008	22	895.89	547.52	1987–2008	22	5.56
Mediterranean countries														
Cyprus	1990–2008	19	25.77 (1.15)	1987–2008	14	76.36	80.70	1999–2008	10	752.23	519.37	1987–2008	22	7.83
France	1987–2008	22	28.45 (0.19)	1987–2008	22	75.13	82.93	1987–2008	22	850.94	453.08	1987–2008	22	5.27
Greece	1987–2008	22	33.40 (0.32)	1987–2008	22	75.70	80.64	1987–2008	22	836.24	576.05	1987–2008	22	6.97
Italy	1987–2008	22	32.91 (0.33)	1987–2008	20	75.66	81.92	1987–2008	20	849.39	501.79	1987–2008	22	6.01
Malta	2000–2008	9	27.97 (0.68)	1987–2008	22	75.27	79.87	1987–2008	22	911.89	619.11	1987–2008	22	8.87
Portugal	1987–2008	22	33.76 (0.69)	1987–2008	20	72.42	79.44	1987–2008	20	1046.94	623.32	1987–2008	22	7.60
Spain	1987–2008	22	32.26 (0.34)	1987–2008	22	75.34	82.37	1987–2008	22	848.94	478.96	1987–2008	22	5.56
Western Balkans														
Albania	1996–2008	13	30.25 (1.03)	1987–2004	17	71.46	77.89	1987–2004	17	1131.47	665.42	1987–2008	22	28.41
Bosnia–Hercegovina	1991–2007	17	32.72 (1.41)	1987–1991	5	69.51	75.19	–	–	–	–	1987–2008	22	16.79
Croatia	1987–2008	22	28.71 (0.69)	1987–2008	22	69.64	77.30	1987–2008	22	1326.29	775.23	1987–2008	22	8.62
Montenegro	2000–2008	9	31.22 (1.03)	1987–2008	20	72.24	78.16	1987–2008	20	1050.29	695.24	1987–2008	22	13.89
Serbia	2002–2008	7	31.14 (1.04)	1994–2008	13	69.98	75.28	1998–2008	11	1303.84	913.33	1987–2008	22	16.47
Slovenia	1987–2008	22	21.38 (0.56)	1987–2008	22	71.54	79.33	1987–2008	22	1153.23	639.76	1987–2008	22	6.00
TFYR Macedonia	1989–2008	20	33.56 (0.90)	1987–2003	14	70.30	75.04	1991–2003	13	1224.10	864.51	1987–2008	22	22.61
Central and Eastern Europe														
Bulgaria	1987–2008	22	26.18 (0.83)	1987–2008	22	68.34	75.30	1987–2008	22	1409.00	894.99	1987–2008	22	13.83
Czech Republic	1987–2008	22	23.96 (0.59)	1987–2008	22	70.75	77.72	1987–2008	22	1264.35	749.67	1987–2008	22	6.27
Hungary	1987–2008	22	28.30 (0.51)	1987–2008	22	66.94	75.64	1987–2008	22	1517.29	844.49	1987–2008	22	10.37
Poland	1987–2008	22	28.69 (0.41)	1987–2008	22	68.76	77.43	1987–2008	22	1346.58	738.32	1987–2008	22	11.30
Romania	1989–2008	20	27.68 (0.86)	1987–2008	22	67.10	74.22	1987–2008	22	1426.36	926.30	1987–2008	22	20.24
Slovakia	1987–2008	22	22.91 (0.82)	1987–2008	22	68.92	77.27	1987–2008	22	1353.97	753.51	1987–2008	22	9.50
Former Soviet Union														
Armenia	1987–2008	22	34.60 (1.98)	1987–2008	21	67.07	73.31	1987–2008	18	1306.84	886.44	1987–2008	22	37.10
Azerbaijan	1987–2008	22	32.82 (2.74)	2007	1	71.33	76.25	1987–2007	19	1316.38	834.21	1987–2008	22	64.45
Belarus	1987–2008	22	25.69 (0.84)	1987–2007	20	64.09	75.02	1987–2007	20	1745.40	886.54	1987–2008	22	12.51
Estonia	1987–2008	22	32.37 (1.13)	1987–2008	22	65.05	76.06	1987–2008	22	1625.19	806.74	1987–2008	22	11.73
Georgia	1987–2008	22	39.73 (2.04)	1987–2006	20	66.56	74.10	1987–2001	14	1217.00	734.36	1987–2008	22	36.48
Latvia	1987–2008	22	29.94 (1.13)	1987–2008	22	64.14	75.18	1987–2008	22	1687.41	850.63	1987–2008	22	11.74
Lithuania	1987–2008	22	30.67 (0.95)	1987–2008	22	65.58	76.58	1987–2008	22	1516.67	760.50	1987–2008	22	10.49
Republic of Moldova	1987–2008	22	35.74 (1.53)	1987–2008	22	64.24	71.57	1987–2008	22	1771.57	1141.94	1987–2008	22	23.84
Russian Federation	1987–2008	22	37.36 (1.53)	1987–2008	22	60.60	72.85	1987–2008	22	1991.94	983.64	1987–2008	22	19.29
Ukraine	1987–2008	22	29.14 (1.69)	1987–2008	21	63.08	73.20	1987–2008	21	1792.63	959.86	1987–2008	22	15.62

^a^N: number of available observations in the study period; SD: standard error of the Gini index within each country over time

**Fig. 1:**
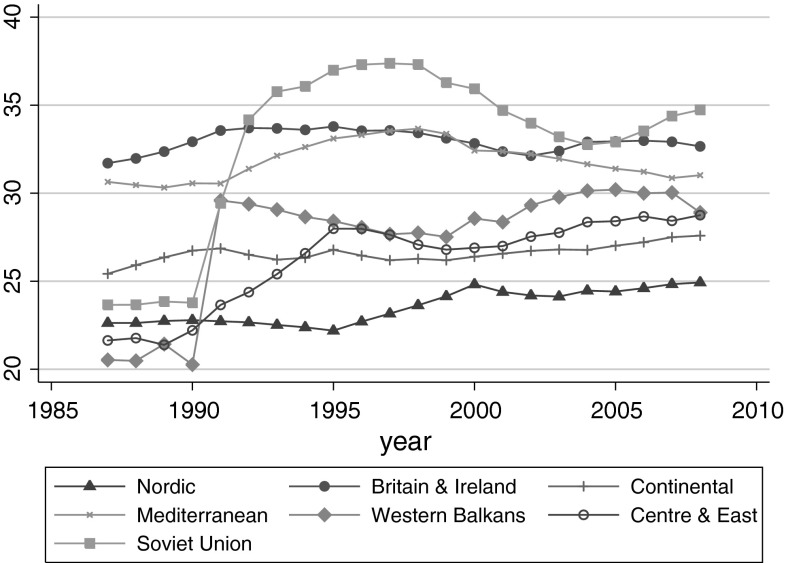
Trends in income inequality (mean GINI) for 7 European regions (41 countries), 1987–2008 (N = 840), the SWIID database. After 1990, UK experienced an increase of income inequality and Ireland experienced a decrease of income inequality. The line for Britain and Ireland is an average trend of these two countries. 41 European countries were included in this graph, where Cyprus and TFYR Macedonia were excluded. This is because the Gini index of Cyprus was much lower than that of other Mediterranean countries and it was available from 1990. The inclusion of Cyprus would cause a sudden decrease of the Mediterranean average Gini index at the point of 1990. Similarly, the Gini index of TFYR Macedonia was much higher than that of other Western Balkan countries and it was available from 1989. The inclusion of TFYR Macedonia would cause a sudden increase of the Western Balkan average Gini index at the point of 1989

Figure 1 shows the trends of the Gini index experienced by the seven European regions over this period. Britain and Ireland and the Mediterranean countries maintained a high level of income inequality over time, while Nordic countries maintained a relatively low level of income inequality. Most regions experienced an increasing trend of income inequality between 1987 and 2008, with a substantial increase in former Soviet countries in the early 1990’s.

Table 2 shows the results of linear regression analyses linking income inequality to life expectancy or cause-specific mortality by gender when all country-year observations were pooled together. Income inequality was significantly and negatively related to life expectancy for both men and women, indicating shorter life expectancies with larger income inequalities. This negative association was stronger for men than for women, and remained significant after additional adjustments for indicators of education, democracy, independence, armed conflicts and economic freedom. For cause-specific mortality, adjusted for GDP and time, income inequality was positively related to mortality from cerebrovascular disease (among women), all infectious disease, signs, symptoms and ill-defined conditions, and homicide (among men), and income inequality was also negatively related to some causes of mortality, such as all cancers and suicide. After further adjustment for indicators of education, democracy, independence, armed conflicts and economic freedom, positive associations with income inequality were found for almost all causes of mortality (except deaths from lung cancer among women, breast cancer, chronic liver diseases and suicide). The inverse association between income inequality and infant mortality was significant and remained significant after adjustment for the potential confounding variables.

**Table 2:** Linear regression coefficients of GINI from pooled cross-sectional analyses linking income inequality and population health measured by life expectancy and mortality, pooled 43 European countries 1987–2008

Outcomes	**Men**		**Women**	
	Model 1^a^	Model 2^b^	Model 1	Model 2
Life expectancy	**−0.0820** **(−0.130, −0.034)**	**−0.2590** **(−0.317, −0.201)**	**−0.0371** **(−0.065, −0.010)**	**−0.0901** **(−0.122, −0.058)**
All causes	0.0001 (−0.003, 0.004)	**0.0128** **(0.009, 0.017)**	−0.0014 (−0.004, 0.001)	**0.0056** **(0.003, 0.008)**
All circulatory disease	−0.0030 (−0.007, 0.001)	**0.0109** **(0.005, 0.016)**	−0.0020 (−0.006, 0.002)	**0.0110** **(0.006, 0.016)**
Ischemic heart disease	0.0022 (−0.005, 0.009)	**0.0156** **(0.007, 0.024)**	0.0006 (−0.007, 0.009)	**0.0119** **(0.003, 0.021)**
Cerebrovascular disease	0.0056 (−0.001, 0.012)	**0.0203** **(0.013, 0.027)**	**0.0098** **(0.004, 0.016)**	**0.0235** **(0.017, 0.030)**
All cancers	**−0.0066** **(−0.010, −0.003)**	**0.0045** **(0.002, 0.007)**	**−0.0055** **(−0.010, −0.001)**	**0.0102** **(0.007, 0.014)**
Cancer of lung	−0.0058 (−0.012, 0.00002)	**0.0148** **(0.010, 0.020)**	**−0.0197** **(−0.026, −0.013)**	**−0.0213** **(−0.030, −0.012)**
Cancer of breast			0.0015 (−0.003, 0.006)	0.0032 (−0.001, 0.007)
All infectious disease	**0.0349** **(0.026, 0.043)**	**0.0463** **(0.036, 0.057)**	**0.0205** **(0.014, 0.027)**	**0.0130** **(0.006, 0.020)**
Chronic liver disease and cirrhosis	0.0003 (−0.013, 0.014)	0.0006 (−0.021, 0.022)	0.0119 (−0.001, 0.025)	0.0034 (−0.014, 0.021)
All external causes	−0.0017 (−0.011, 0.008)	**0.0301** **(0.020, 0.041)**	**−0.0086** **(−0.017, −0.001)**	**0.0162** **(0.008, 0.024)**
Motor vehicle accidents	0.0053 (−0.004, 0.015)	**0.0381** **(0.029, 0.048)**	0.0010 (−0.008, 0.010)	**0.0293** **(0.020, 0.038)**
Suicide	**−0.0375** **(−0.053, −0.022)**	0.0070 (−0.006, 0.020)	**−0.0443** **(−0.057, −0.031)**	**−0.0139** **(−0.026, −0.002)**
Signs, symptoms and ill-defined	**0.0249** **(0.008, 0.042)**	**0.0455** **(0.21, 0.070)**	**0.0234** **(0.004, 0.042)**	**0.0377** **(0.011, 0.065)**
Homicide	**0.0175** **(0.001, 0.034)**	**0.0584** **(0.039, 0.078)**	−0.0089 (−0.024, 0.006)	**0.0256** **(0.008, 0.043)**
	**Infant**			
Infant mortality	**0.0216** **(0.015, 0.028)**	**0.0166** **(0.012, 0.021)**		

Bold values indicate significant results at the 95 % confidence level

^a^Model 1 includes Gini index (95 % confidence interval in parentheses, based on robust standard errors), year dummies and log (gdp)

^b^Model 2 additionally adds democracy index, education, independence, armed conflict and economic freedom

Table 3 presents the results from fixed effects models linking changes in income inequality to changes in life expectancy or cause-specific mortality. All associations between income inequality and mortality indicators became insignificant, except for death from external causes and homicide among men, and all cancers among women. The significant results of homicide and all cancers disappeared after further adjustment of more country characteristics. In further analyses (Supplementary Table 2), the positive relation between income inequality and death from external causes appeared not robust to variations in model specifications.

**Table 3:** Linear regression coefficients of GINI from fixed effects models linking income inequality and population health measured by life expectancy and mortality, 43 European countries 1987–2008

Outcomes	**Men**		**Women**	
	Model 1^a^	Model 2^b^	Model 1	Model 2
Life expectancy	−0.0754 (−0.178, 0.027)	−0.0811 (−0.174, 0.012)	−0.0049 (−0.071, 0.061)	−0.0361 (−0.077, 0.005)
All causes	0.0054 (−0.0005, 0.011)	0.0044 (−0.001, 0.010)	0.00002 (−0.005, 0.005)	0.0020 (−0.002, 0.006)
All circulatory diseases	0.0063 (−0.003, 0.015)	0.0022 (−0.005, 0.010)	0.0022 (−0.006, 0.011)	0.0010 (−0.004, 0.006)
Ischemic heart disease	0.0060 (−0.003, 0.015)	−0.0009 (−0.011, 0.009)	0.0019 (−0.007, 0.011)	−0.0044 (−0.015, 0.006)
Cerebrovascular disease	0.0061 (−0.004, 0.017)	0.0036 (−0.005, 0.013)	0.0029 (−0.008, 0.014)	0.0034 (−0.005, 0.011)
All cancers	0.0035 (−0.001, 0.008)	0.0032 (−0.0001, 0.007)	**0.0051** **(0.0001, 0.010)**	0.0036 (−0.001, 0.009)
Cancer of lung	0.0030 (−0.002, 0.008)	0.0018 (−0.006, 0.009)	−0.0068 (−0.016, 0.003)	−0.0052 (−0.016, 0.005)
Cancer of breast			0.0061 (−0.003, 0.015)	0.0035 (−0.003, 0.010)
All infectious diseases	0.0068 (−0.008, 0.022)	0.0235 (−0.009, 0.056)	0.00005 (−0.015, 0.015)	0.0126 (−0.013, 0.038)
Chronic liver disease and cirrhosis	0.0153 (−0.004, 0.035)	0.0086 (−0.015, 0.032)	0.0153 (−0.007, 0.037)	0.0015 (−0.021, 0.024)
All external causes	**0.0143** **(0.0002, 0.028)**	**0.0143** **(0.001, 0.028)**	0.0078 (−0.007, 0.023)	**0.0158** **(0.001, 0.031)**
Motor vehicle accidents	0.0091 (−0.007, 0.025)	0.0048 (−0.009, 0.018)	0.0109 (−0.004, 0.026)	0.0054 (−0.009, 0.020)
Suicide	0.0051 (−0.007, 0.017)	0.0104 (−0.0003, 0.021)	−0.0009 (−0.015, 0.013)	0.0127 (−0.001, 0.027)
Signs, symptoms and ill-defined	0.0219 (−0.011, 0.055)	0.0338 (−0.003, 0.070)	0.0078 (−0.029, 0.045)	0.0290 (−0.007, 0.065)
Homicide	**0.0285** **(0.008, 0.049)**	0.0171 (−0.002, 0.036)	0.0147 (−0.004, 0.033)	0.0066 (−0.009, 0.022)
	**Infant**			
Infant mortality	−0.0023 (−0.007, 0.002)	−0.0498 (−0.130, 0.031)		

Bold values indicate significant results at the 95 % confidence level

^a^Model 1 includes Gini index (95 % confidence interval in parentheses, based on clustered standard errors), year dummies, log (gdp) and country fixed effects

^b^Model 2 additionally adds democracy index, education, independence, armed conflict and economic freedom

Essentially similar results were obtained in supplementary analyses for robustness checks: allowing a different time trend and interactive effects of the country characteristics for the former Soviet countries, allowing country-specific linear time trends, restricting the analyses to high-income countries, and using gross income Gini index. Moreover, we also introduced up to 10 year lags between the income inequality and life expectancy or infant mortality into the fixed effects models. None of the lagged terms of Gini index was significant. Simultaneously controlling all preceding income inequalities [33] gave essentially similar results (available upon request). Similar analyses were conducted using interpolated LIS data, which also have good quality but much less country-year observations. Again, statistically significant associations between income inequality and mortality were rare. Additionally controlling for the unemployment rate did not change our main findings (results not shown). These checks indicate that our main findings are robust against different model specifications, sample changes, using a Gini index based on gross household income and using another dataset for income inequality.

## Discussion

### Summary of main findings

Significant associations between income inequality and many mortality indicators were found in pooled cross-sectional regressions, indicating higher mortality in countries with larger income inequalities. However, once the country fixed effects were added, all associations between income inequality and mortality indicators became insignificant, except for all external causes and homicide among men, and all cancers among women. The significant results of homicide and all cancers disappeared after further adjustment for indicators of democracy, average years of schooling, transition to national independence, armed conflicts, and economic freedom.

### Study limitations

To identify the link between income inequality and mortality, we adjusted for an array of country characteristics. In order to be confounding variables, these factors should be related to both income inequality and mortality. To the extent however, that these indicators result from income inequality, and therefore should be considered as mediating variables, we may have over-controlled the analyses and thereby removed part of the association between income inequality on mortality. Whether the country characteristic should be seen as a confounder or mediator is not easy to determine, especially for education [34]. On the one hand, investing in education could be a strategy to reduce income inequality within a country [30], making it a potential confounder. On the other hand, high levels of income inequality and the associated underinvestment in public resources might in the long run lead to lower levels of education [35], which makes it a potential mediator. However, most associations between income inequality and mortality indicators were insignificant in the fixed effects models even without controlling for education and other country time-variant characteristics. Thus, the general conclusions are not threatened by the potential problem of “over-controlling”.

In the main analyses, we related annual changes in income inequality to simultaneous annual changes in mortality. We also investigated the effects of income inequality in a specific year on mortality up to 10 years later, which produced similar results. This approach may be insufficient to fully capture the cumulative impact of a history of large income inequalities on mortality [21, 22, 36]. Future research, using data over even longer time-periods than available in our study, are necessary to explore the effects of long-term exposure to income inequality.

Our analysis was limited to European countries. This reduced the potential for confounding as country-level confounding variables can be expected to be more similar in Europe than in a global context. It also produced results that are relevant for policy makers in European countries, who are likely to be more concerned with the effect of income inequality as observed within the range of variation prevailing in a European context, than with the more extreme values of income inequality observed elsewhere in the world. However, it is important to note that our results cannot be generalized beyond this smaller range of variation, and that analyses including countries outside Europe with larger increases in income inequality over time may lead to different conclusions.

### Interpretation

Our findings are in line with previous studies that found negative cross-sectional associations between income inequality and population health [1, 15, 16], and with some existing studies using fixed effects models where no significant effects were found [6, 19, 21]. We further strengthened the evidence however, because our results were obtained in a study in which we focused on a large array of European countries over a relatively long period of time, used better data on income inequalities and a set of disease-specific mortality indicators, considered a larger set of potential confounding variables, and used a country fixed effects approach. The only significant result in our fixed effects analysis, after adjustment for country characteristics, was the association between income inequality and external causes mortality. This cause of death group is strongly related to individual socioeconomic status [37, 38]. However, the results for all external causes mortality were not robust to variations in model specifications in further analyses (Supplementary Table 2).

Differences between results from pooled cross-sectional analyses and fixed effects models indicate that the observed association between income inequality and mortality is likely to result from confounding, and that income inequality as such is not a driving force of poor population health. We can only speculate which country characteristics might be responsible for the disappearance of the effect, and suspect that these are historical, social or cultural factors that are associated with both the hierarchical nature of societies, as indicated by income inequality, and the health of their populations. Unfortunately, many of these factors are not available in international databases. Further research is necessary to find appropriate measures for the relevant country characteristics and test their effects on the association between income inequality and mortality.

One underlying factor determining both income inequality and mortality could be social and health policies that vary across countries and are persistent over time. For example, poverty reduction policies such as minimum wage, disability allowances and return to work programs can reduce income inequality and simultaneously improve average population health by improving health of the poorest part of the population. Besides these, health care programs such as smoking cessation strategies, maternal education programs and cancer screening may also play roles since they tend to cluster in countries with strong redistribution policies, although without having a direct impact on income inequality [21]. The implementation of all these policies has varied between European countries [39], which could have produced a “spurious” association between income inequality and mortality. Other responsible factors could be some cultural and historical elements of a country, e.g. egalitarianism (importance of transcending self-interest and promoting the welfare of others), power distance (extent to which the less powerful accept that power is distributed unequally) and ethnic heterogeneity, which are potentially important determinants of population health [40, 41], and at the same time could be related to income inequality [42, 43]. The disappearance of the association between income inequality and health when moving to fixed effects models could be the result of controlling for these country heterogeneities.

It has been noted that the most consistent evidence for an adverse effect of income inequality on population health derives from within-country differences in the United States or some other countries with comparable or even larger income inequalities [3, 36]. The studies from countries having more equal income distribution outside of Europe, e.g. Canada, Australia and Japan, often produced insignificant findings [44, 45]. Therefore, one possible explanation for not finding significant relationships in our study is that most European countries in our sample are more egalitarian than the United States. It has been suggested that there is a “threshold effect”, implying the existence of a threshold of income inequality above which adverse impacts on health begin to emerge [46]. However, this appears to only partly explain our findings, since restricting the analysis to the 18 countries with a mean Gini index larger than 30 (a potential threshold value suggested in the literature [46] ) still produced insignificant results (results not shown). Another possible explanation for not finding significant relationships is that income inequality may be less strongly associated with the social distribution of major risk factors in Europe. For example, the delay of the epidemiologic transition and the more egalitarian social distribution of healthy “Mediterranean diets” in southern Europe make lower income less a risk factor for cardiovascular disease mortality in these countries [3, 47, 48]. Meanwhile, the well-developed welfare system in Europe, especially in some northern and continental European countries, may help to buffer the adverse effect on mortality of being poor [49]. A deeper exploration of why income inequality does not have more effect in Europe is needed.

## Conclusions

Within Europe, cross-sectional associations between income inequality and mortality probably result from confounding. Fixed effect models which remove time-invariant country heterogeneity suggest that there is no statistically significant relation between income inequality and population health measured by life expectancy and cause-specific mortality in European countries between 1987 and 2008. Although reducing income inequality may be important for creating equality of opportunity and for the reduction of health inequalities, it has a limited role for reducing average mortality in Europe.

## Electronic supplementary material

Supplementary material 1 (DOCX 53 kb)
