# Supplementary material for: Income inequality, life expectancy and cause-specific mortality in 43 European countries, 1987–2008: a fixed effects study
Source: Eur J Epidemiol. 2015 Jul 16;30(8):615–25. doi: 10.1007/s10654-015-0066-x (PMC4579249; doi:10.1007/s10654-015-0066-x)
Supplement: Supplementary file 1 — Supplementary material 1 (DOCX 53 kb) [file 10654_2015_66_MOESM1_ESM.docx]

**Supplementary material**

**Supplementary Table 1** Descriptive statistics of the other variables used in the analysis

|  | (1) | (2) | (3) | (4) | (5) |
| --- | --- | --- | --- | --- | --- |
| VARIABLES | N | mean | sd | min | max |
|  |  |  |  |  |  |
| democracy | 932 | 6.668 | 5.494 | -9 | 10 |
| gdp | 934 | 12,136 | 7,997 | 1,632 | 53,962 |
| education | 814 | 9.632 | 1.323 | 5.735 | 13.09 |
| conflict | 946 | 0.122 | 0.513 | 0 | 3 |
| independence | 946 | 0.079 | 0.270 | 0 | 1 |
| economic free | 724 | 6.609 | 1.219 | 2.775 | 8.390 |
| male_all | 848 | 1,181 | 354.6 | 595.1 | 2,382 |
| male_cir | 825 | 554.1 | 236.0 | 165.7 | 1,184 |
| male_isch | 824 | 276.8 | 168.4 | 53.28 | 737.6 |
| male_cere | 825 | 137.2 | 75.13 | 30.92 | 379.4 |
| male_neo | 825 | 183.0 | 33.33 | 75.22 | 278.0 |
| male_lung | 824 | 69.46 | 20.80 | 16.95 | 120.4 |
| male_infe | 824 | 13.37 | 9.832 | 1.170 | 61.07 |
| male_liver | 748 | 26.97 | 22.97 | 0 | 128.4 |
| male_exte | 825 | 110.6 | 77.88 | 26.48 | 459.9 |
| male_traf | 793 | 20.14 | 10.51 | 1.430 | 68.79 |
| male_suic | 818 | 26.10 | 17.55 | 1.160 | 89.27 |
| male_sign | 799 | 36.08 | 42.95 | 0.340 | 346.7 |
| male_homi | 827 | 6.393 | 10.74 | 0 | 106.4 |
| fe_all | 848 | 697.5 | 181.7 | 380.5 | 1,358 |
| fe_cir | 825 | 362.7 | 160.5 | 96.43 | 758.9 |
| fe_isch | 824 | 150.5 | 107.1 | 12.80 | 517.4 |
| fe_cere | 825 | 109.7 | 57.98 | 23.42 | 277.0 |
| fe_neo | 825 | 249.3 | 52.22 | 93.85 | 391.1 |
| fe_resp | 824 | 14.51 | 8.448 | 3.240 | 44.54 |
| fe_brea | 825 | 25.95 | 6.601 | 6.020 | 53.99 |
| fe_infe | 824 | 5.657 | 2.725 | 0.300 | 19.62 |
| fe_liver | 748 | 11.59 | 14.87 | 0 | 105.1 |
| fe_exte | 825 | 34.21 | 25.95 | 7.890 | 611.1 |
| fe_traf | 793 | 5.762 | 2.703 | 0 | 16.11 |
| fe_suic | 818 | 6.842 | 3.835 | 0 | 23.18 |
| fe_sign | 799 | 23.52 | 34.57 | 0.210 | 291.5 |
| fe_homi | 827 | 2.019 | 2.391 | 0 | 14.33 |
|  |  |  |  |  |  |

**Supplementary Table 2**  Linear regression coefficients of GINI from fixed effects models (different specifications) linking income inequality and population health measured by life expectancy and mortality,

43 European countries 1987-2008

| **Outcomes** | **Men** | | |  | **Women** | | |
| --- | --- | --- | --- | --- | --- | --- | --- |
|  | Model 1^a^ | Model 2^b^ | Model 3^c^ |  | Model 1 | Model 2 | Model 3 |
| Life expectancy | -0.0194 | -0.0371 | -0.0281 |  | 0.0136 | -0.0169 | 0.0021 |
|  | (-0.099, 0.060) | (-0.088, 0.014) | (-0.113, 0.057) |  | (-0.040, 0.067) | (-0.048, 0.014) | (-0.068, 0.072) |
| All causes | 0.0027 | 0.0021 | 0.0008 |  | -0.0018 | 0.0006 | -0.0007 |
|  | (-0.002, 0.007) | (-0.001, 0.006) | (-0.003, 0.004) |  | (-0.005, 0.001) | (-0.002, 0.004) | (-0.004, 0.002) |
| All circulatory diseases | 0.0020 | 0.0014 | -0.0006 |  | -0.001 | 0.0014 | -0.0012 |
|  | (-0.004, 0.007) | (-0.004, 0.007) | (-0.003, 0.002) |  | (-0.006, 0.004) | (-0.003, 0.005) | (-0.004, 0.002) |
| Ischemic heart disease | 0.0025 | 0.0002 | -0.0005 |  | 0.0009 | -0.0014 | -0.0024 |
|  | (-0.006, 0.011) | (-0.009, 0.010) | (-0.005, 0.004) |  | (-0.008, 0.010) | (-0.012, 0.009) | (-0.009, 0.004) |
| Cerebrovascular disease | 0.0021 | 0.0011 | -0.0008 |  | -0.0003 | 0.0015 | -0.0009 |
|  | (-0.005, 0.009) | (-0.010, 0.012) | (-0.006, 0.004) |  | (-0.008, 0.008) | (-0.008, 0.011) | (-0.006, 0.004) |
| All cancers | 0.0028 | 0.0036 | -0.0019 |  | 0.0043 | 0.0044 | -0.0018 |
|  | (-0.001, 0.007) | (-0.001, 0.008) | (-0.006, 0.003) |  | (-0.0002, 0.009) | (-0.001, 0.010) | (-0.006, 0.003) |
| Cancer of lung | 0.0029 | 0.0039 | -0.00317 |  | -0.0027 | -0.0016 | -0.0045 |
|  | (-0.002, 0.008) | (-0.004, 0.012) | (-0.009, 0.003) |  | (-0.008, 0.003) | (-0.008, 0.005) | (-0.012, 0.003) |
| Cancer of breast |  |  |  |  | 0.0033 | 0.0015 | -0.0015 |
|  |  |  |  |  | (-0.002, 0.009) | (-0.006, 0.009) | (-0.008, 0.005) |
| All infectious diseases | 0.0058 | 0.0228 | -0.0018 |  | 0.0021 | 0.0119 | -0.0008 |
|  | (-0.010, 0.021) | (-0.006, 0.052) | (-0.011, 0.007) |  | (-0.012, 0.017) | (-0.012, 0.036) | (-0.010, 0.009) |
| Chronic liver disease and cirrhosis | 0.0092 | 0.0075 | 0.0043 |  | 0.0077 | -0.0019 | 0.0056 |
|  | (-0.005, 0.024) | (-0.017, 0.032) | (-0.006, 0.015) |  | (-0.007, 0.022) | (-0.025, 0.022) | (-0.004, 0.016) |
| All external causes | 0.0122 | 0.0069 | 0.0065 |  | 0.0059 | **0.0105** | 0.0004 |
|  | (-0.003, 0.027) | (-0.002, 0.016) | (-0.006, 0.019) |  | (-0.009, 0.021) | **(0.002, 0.019)** | (-0.013, 0.013) |
| Motor vehicle accidents | 0.0123 | 0.0046 | 0.0020 |  | 0.0109 | 0.0053 | -0.0009 |
|  | (-0.005, 0.029) | (-0.009, 0.018) | (-0.011, 0.015) |  | (-0.006, 0.028) | (-0.008, 0.019) | (-0.013, 0.012) |
| Suicide | 0.0040 | 0.0057 | -0.0101 |  | -0.0003 | 0.0107 | -0.0147 |
|  | (-0.007, 0.015) | (-0.005, 0.016) | (-0.030, 0.010) |  | (-0.014, 0.014) | (-0.004, 0.025) | (-0.039, 0.009) |
| Signs, symptoms and ill-defined | 0.0193 | 0.0251 | 0.0112 |  | 0.0082 | 0.0207 | 0.0146 |
|  | (-0.012, 0.050) | (-0.013, 0.063) | (-0.011. 0.033) |  | (-0.027, 0.044) | (-0.017, 0.059) | (-0.012, 0.041) |
| Homicide | **0.0257** | 0.0087 | 0.0038 |  | 0.0130 | 0.0006 | -0.0085 |
|  | **(0.002, 0.049)** | (-0.009, 0.026) | (-0.013, 0.020) |  | (-0.008, 0.034) | (-0.012, 0.014) | (-0.024, 0.007) |
|  | **Infant** | | |  |  |  |  |
| Infant mortality | -0.0027 | -0.0043 | 0.0005 |  |  |  |  |
|  | (-0.007, 0.002) | (-0.012, 0.004) | (-0.003, 0.004) |  |  |  |  |

Notes:

a. Model 1 includes Gini index (95% confidence interval in parentheses, based on clustered standard errors), year dummies, log(gdp), year*soviet dummy and country fixed effects. Soviet dummy is 1 for Armenia, Azerbaijan, Belarus, Estonia, Georgia, Latvia, Lithuania, Republic of Moldova, Russian Federation and Ukraine, and soviet dummy is 0 for all other countries.

b. Model 2 additionally adds democracy index, education, independence, armed conflict, economic freedom, and the interactions between the soviet dummy and the adjusted variables.

c. Model 3 includes Gini index, year dummies, country fixed effects, log(gdp) and country-specific linear time trends.

**Supplementary Table 3**  Linear regression coefficients of GINI from fixed effects models linking income inequality and population health measured by life expectancy and mortality,

19 high-income European countries^a^ 1987-2008

| **Outcomes** | **Men** | |  | **Women** | |
| --- | --- | --- | --- | --- | --- |
|  | Model 1^b^ | Model 2^c^ |  | Model 1 | Model 2 |
| Life expectancy | 0.0055 | 0.0105 |  | 0.0414 | 0.0406 |
|  | (-0.051, 0.061) | (-0.038, 0.059) |  | (-0.011, 0.094) | (-0.014, 0.095) |
| All causes | -0.0004 | -0.0008 |  | -0.0038 | -0.0038 |
|  | (-0.006, 0.005) | (-0.005, 0.003) |  | (-0.009, 0.002) | (-0.009, 0.002) |
| All circulatory diseases | -0.0005 | -0.0008 |  | -0.0007 | -0.0006 |
|  | (-0.008, 0.007) | (-0.007, 0.005) |  | (-0.008, 0.006) | (-0.008, 0.006) |
| Ischemic heart disease | 0.0034 | 0.0021 |  | 0.0036 | 0.0031 |
|  | (-0.013, 0.020) | (-0.016, 0.020) |  | (-0.016, 0.023) | (-0.018, 0.024) |
| Cerebrovascular disease | -0.0061 | -0.0064 |  | -0.0067 | -0.0069 |
|  | (-0.022, 0.010) | (-0.023, 0.010) |  | (-0.021, 0.007) | (-0.022, 0.008) |
| All cancers | 0.0032 | 0.0027 |  | 0.0045 | 0.0037 |
|  | (-0.006, 0.012) | (-0.004, 0.009) |  | (-0.007, 0.016) | (-0.004, 0.011) |
| Cancer of lung | 0.0039 | 0.0033 |  | -0.0079 | -0.0058 |
|  | (-0.016, 0.024) | (-0.012, 0.018) |  | (-0.021, 0.005) | (-0.015, 0.004) |
| Cancer of breast |  |  |  | -0.0003 | -0.0003 |
|  |  |  |  | (-0.016, 0.016) | (-0.015, 0.014) |
| All infectious diseases | 0.0481 | 0.0483 |  | 0.0304 | 0.0317 |
|  | (-0.003, 0.099) | (-0.005, 0.102) |  | (-0.016, 0.077) | (-0.019, 0.082) |
| Chronic liver disease and cirrhosis | 0.0049 | 0.0037 |  | -0.0103 | -0.0101 |
|  | (-0.042, 0.052) | (-0.039, 0.047) |  | (-0.057, 0.037) | (-0.054, 0.033) |
| All external causes | 0.0058 | 0.0054 |  | 0.0148 | **0.0148** |
|  | (-0.009, 0.021) | (-0.008, 0.019) |  | (-0.0002, 0.030) | **(0.003, 0.027)** |
| Motor vehicle accidents | 0.0134 | 0.0126 |  | 0.0142 | 0.0132 |
|  | (-0.012, 0.039) | (-0.013, 0.038) |  | (-0.011, 0.039) | (-0.012, 0.039) |
| Suicide | 0.0023 | 0.0011 |  | 0.0139 | 0.0137 |
|  | (-0.019, 0.023) | (-0.019, 0.021) |  | (-0.020, 0.048) | (-0.019, 0.046) |
| Signs, symptoms and ill-defined | 0.0074 | 0.0046 |  | -0.0032 | -0.0055 |
|  | (-0.051, 0.066) | (-0.058, 0.067) |  | (-0.062, 0.055) | (-0.067, 0.056) |
| Homicide | -0.0076 | -0.0068 |  | 0.0023 | 0.0033 |
|  | (-0.037, 0.021) | (-0.035, 0.021) |  | (-0.021, 0.026) | (-0.022, 0.028) |
|  | **Infant** | |  |  |  |
| Infant mortality | **-0.0170** | **-0.0172** |  |  |  |
|  | **(-0.033, -0.002)** | **(-0.031, -0.003)** |  |  |  |

Notes:

a. Countries included in the analysis are Austria, Belgium, Denmark, Finland, France, Germany, Greece, Iceland, Ireland, Italy, Luxembourg, Malta, Netherlands, Norway, Portugal, Spain, Sweden, Switzerland and United Kingdom.

b. Model 1 includes Gini index (95% confidence interval in parentheses, based on clustered standard errors), year dummies, log(gdp) and country fixed effects.

c. Model 2 additionally adds democracy index, education, independence, armed conflict and economic freedom.

**Supplementary** **Table 4**  Linear regression coefficients of GINI (based on gross income) from fixed effects models linking income inequality and population health measured by life expectancy and mortality,

43 European countries 1987-2008

| **Outcomes** | **Men** | |  | **Women** | |
| --- | --- | --- | --- | --- | --- |
|  | Model 1^a^ | Model 2^b^ |  | Model 1 | Model 2 |
| Life expectancy | -0.0185 | -0.0283 |  | 0.0055 | -0.0143 |
|  | (-0.086, 0.049) | (-0.077, 0.021) |  | (-0.036, 0.047) | (-0.039, 0.010) |
| All causes | 0.0017 | 0.0017 |  | -0.0006 | 0.0011 |
|  | (-0.002, 0.006) | (-0.002, 0.005) |  | (-0.004, 0.002) | (-0.001, 0.004) |
| All circulatory diseases | 0.0009 | 0.0002 |  | -0.0006 | 0.0003 |
|  | (-0.005, 0.007) | (-0.005, 0.005) |  | (-0.006, 0.004) | (-0.003, 0.004) |
| Ischemic heart disease | 0.0007 | -0.0003 |  | -0.0001 | -0.0009 |
|  | (-0.006, 0.007) | (-0.007, 0.007) |  | (-0.007, 0.007) | (-0.009, 0.007) |
| Cerebrovascular disease | 0.0015 | 0.0030 |  | 0.0006 | 0.0036 |
|  | (-0.006, 0.009) | (-0.004, 0.010) |  | (-0.007, 0.008) | (-0.002, 0.009) |
| All cancers | 0.0017 | 0.0021 |  | 0.0026 | 0.0025 |
|  | (-0.001, 0.005) | (-0.001, 0.005) |  | (-0.001, 0.006) | (-0.0009, 0.006) |
| Cancer of lung | 0.0028 | 0.0036 |  | -0.0010 | 0.0006 |
|  | (-0.001, 0.007) | (-0.001, 0.008) |  | (-0.007, 0.005) | (-0.006, 0.007) |
| Cancer of breast |  |  |  | 0.0026 | 0.0020 |
|  |  |  |  | (-0.003, 0.008) | (-0.002, 0.006) |
| All infectious diseases | 0.0046 | 0.0117 |  | 0.0030 | 0.0076 |
|  | (-0.007, 0.017) | (-0.007, 0.030) |  | (-0.008, 0.014) | (-0.007, 0.022) |
| Chronic liver disease and cirrhosis | 0.0085 | 0.0091 |  | 0.0052 | 0.0004 |
|  | (-0.004, 0.021) | (-0.007, 0.026) |  | (-0.007, 0.017) | (-0.013, 0.014) |
| All external causes | 0.0066 | 0.0054 |  | 0.0046 | **0.0089**** |
|  | (-0.004, 0.018) | (-0.002, 0.013) |  | (-0.006, 0.015) | **(0.0010, 0.017)** |
| Motor vehicle accidents | 0.0043 | 0.0024 |  | 0.0046 | 0.0033 |
|  | (-0.008, 0.016) | (-0.007, 0.012) |  | (-0.007, 0.016) | (-0.006, 0.013) |
| Suicide | 0.0030 | 0.0060 |  | 0.0030 | **0.0110**** |
|  | (-0.006, 0.012) | (-0.002, 0.014) |  | (-0.008, 0.014) | **(0.0002, 0.022)** |
| Signs, symptoms and ill-defined | 0.0150 | 0.0183 |  | 0.0071 | 0.0163 |
|  | (-0.012, 0.042) | (-0.011, 0.047) |  | (-0.023, 0.037) | (-0.014, 0.047) |
| Homicide | 0.0161 | 0.0091 |  | 0.0095 | 0.0046 |
|  | (-0.003, 0.035) | (-0.005, 0.023) |  | (-0.007, 0.026) | (-0.006, 0.015) |
|  | **Infant** | |  |  |  |
| Infant mortality | -0.0029 | -0.0536 |  |  |  |
|  | (-0.007, 0.001) | (-0.119, 0.012) |  |  |  |

Notes:

a. Model 1 includes Gini index based on gross income (95% confidence interval in parentheses, based on clustered standard errors), year dummies, log(gdp) and country fixed effects.

b. Model 2 additionally adds democracy index, education, independence, armed conflict and economic freedom.

**Supplementary Table 5**  Linear regression coefficients of lagged GINI from fixed effects models linking lagged terms of income inequality and population health measured by life expectancy and infant mortality,

43 European countries 1987-2008

|  | **Male life expectancy** | |  | **Female life expectancy** | |  | **Infant mortality** | |
| --- | --- | --- | --- | --- | --- | --- | --- | --- |
|  | Model 1^a^ | Model 2^b^ |  | Model 1 | Model 2 |  | Model 1 | Model 2 |
| lag-1 year | -0.0684 | -0.0692 |  | -0.0046 | -0.0336 |  | -0.0033 | -0.0033 |
|  | (-0.167, 0.030) | (-0.164, 0.025) |  | (-0.070, 0.061) | (-0.077, 0.0099) |  | (-0.009, 0.002) | (-0.011, 0.004) |
| lag-2 years | -0.0613 | -0.0493 |  | -0.0120 | -0.0250 |  | -0.0035 | -0.0032 |
|  | (-0.136, 0.014) | (-0.137, 0.038) |  | (-0.054, 0.030) | (-0.071, 0.0213) |  | (-0.009, 0.002) | (-0.011, 0.005) |
| lag-3 years | -0.0346 | -0.00496 |  | -0.0014 | 0.0028 |  | -0.0031 | -0.0033 |
|  | (-0.092, 0.023) | (-0.072, 0.062) |  | (-0.035, 0.032) | (-0.038, 0.044) |  | (-0.009, 0.0024) | (-0.011, 0.005) |
| lag-4 years | -0.0180 | 0.0366 |  | 0.00045 | 0.0246 |  | -0.0026 | -0.0032 |
|  | (-0.062, 0.026) | (-0.020, 0.093) |  | (-0.026, 0.026) | (-0.013, 0.063) |  | (-0.008, 0.003) | (-0.011, 0.004) |
| lag-5 years | -0.0102 | 0.0235 |  | 0.0007 | 0.0168 |  | -0.0022 | -0.0026 |
|  | (-0.050, 0.029) | (-0.024, 0.071) |  | (-0.022, 0.023) | (-0.019, 0.052) |  | (-0.007, 0.003) | (-0.010, 0.005) |
| lag-6 years | -0.0067 | 0.0020 |  | 0.0065 | 0.0084 |  | -0.0021 | -0.0027 |
|  | (-0.047, 0.033) | (-0.041, 0.045) |  | (-0.013, 0.026) | (-0.022, 0.038) |  | (-0.007, 0.003) | (-0.010, 0.005) |
| lag-7 years | -0.0155 | -0.0214 |  | 0.0022 | -0.0055 |  | -0.0022 | -0.0030 |
|  | (-0.055, 0.024) | (-0.067, 0.025) |  | (-0.020, 0.024) | (-0.038, 0.027) |  | (-0.007, 0.002) | (-0.010, 0.004) |
| lag-8 years | -0.0283 | -0.0402 |  | -0.0037 | -0.0123 |  | -0.0022 | -0.0038 |
|  | (-0.074, 0.017) | (-0.100, 0.019) |  | (-0.032, 0.025) | (-0.056, 0.031) |  | (-0.007, 0.002) | (-0.011, 0.004) |
| lag-9 years | -0.0346 | -0.0461 |  | -0.0091 | -0.0170 |  | -0.0019 | -0.0038 |
|  | (-0.086, 0.016) | (-0.110, 0.018) |  | (-0.041, 0.023) | (-0.066, 0.032) |  | (-0.006, 0.0025) | (-0.011, 0.0033) |
| lag-10 years | -0.0364 | -0.0467 |  | -0.0144 | -0.0232 |  | -0.0012 | -0.0031 |
|  | (-0.089, 0.016) | (-0.108, 0.015) |  | (-0.048, 0.019) | (-0.071, 0.025) |  | (-0.006, 0.0035) | (-0.010, 0.004) |
|  |  | | |  |  |  |  |  |

Notes:

a. Model 1 includes lagged terms of Gini index (95% confidence interval in parentheses, based on clustered standard errors), year dummies, log(gdp) and country fixed effects.

b. Model 2 additionally adds democracy index, education, independence, armed conflict and economic freedom.

**Supplementary Table 6** Linear regression coefficients of GINI (from Luxembourg Income Study) from fixed effects models linking income inequality and population health measured by life expectancy and mortality,

24 European countries for the year 1990/1995/2000/2005^a^

| **Outcomes** | **Men** | |  | **Women** | |
| --- | --- | --- | --- | --- | --- |
|  | Model 1^b^ | Model 2^c^ |  | Model 1 | Model 2 |
| Life expectancy | 2.538 | 2.087 |  | 4.220 | 2.862 |
|  | (-5.123, 10.20) | (-5.559, 9.733) |  | (-3.650, 12.09) | (-5.576, 11.30) |
| All causes | -0.153 | -0.402 |  | -0.360 | -0.405 |
|  | (-0.889, 0.583) | (-0.993, 0.189) |  | (-1.126, 0.407) | (-1.200, 0.391) |
| All circulatory diseases | 0.0351 | -0.668 |  | 0.386 | -0.269 |
|  | (-1.306, 1.376) | (-1.657, 0.322) |  | (-1.043, 1.814) | (-1.438, 0.901) |
| Ischemic heart disease | 0.0714 | -1.424 |  | 1.111 | -0.962 |
|  | (-1.987, 2.130) | (-4.021, 1.174) |  | (-1.273, 3.494) | (-4.118, 2.195) |
| Cerebrovascular disease | -0.582 | -1.940 |  | -0.717 | **-2.033** |
|  | (-2.359, 1.195) | (-3.937, 0.057) |  | (-2.554, 1.120) | **(-3.833, -0.234)** |
| All cancers | 0.217 | -0.244 |  | 0.412 | -0.189 |
|  | (-0.645, 1.079) | (-1.135, 0.646) |  | (-0.616, 1.440) | (-1.207, 0.829) |
| Cancer of lung | -0.458 | -1.060 |  | -0.845 | -0.768 |
|  | (-1.901, 0.984) | (-2.357, 0.238) |  | (-2.204, 0.515) | (-1.954, 0.419) |
| Cancer of breast |  |  |  | 0.610 | -0.0790 |
|  |  |  |  | (-1.008, 2.227) | (-2.087, 1.929) |
| All infectious diseases | 3.125 | 7.182 |  | 0.133 | 3.589 |
|  | (-4.558, 10.81) | (-2.023, 16.39) |  | (-6.105, 6.371) | (-4.827, 12.01) |
| Chronic liver disease and cirrhosis | -0.166 | 0.0147 |  | 2.060 | 2.610 |
|  | (-3.381, 3.049) | (-4.075, 4.104) |  | (-1.366, 5.485) | (-1.375, 6.595) |
| All external causes | 1.066 | 1.401 |  | 1.137 | **2.120** |
|  | (-0.142, 2.273) | (-0.071, 2.873) |  | (-0.333, 2.607) | **(0.834, 3.405)** |
| Motor vehicle accidents | **2.206** | 2.519 |  | **3.811** | **4.028** |
|  | **(0.063, 4.349)** | (-0.023, 5.061) |  | **(2.322, 5.299)** | **(2.121, 5.934)** |
| Suicide | 0.216 | -0.237 |  | -0.546 | -0.648 |
|  | (-1.815, 2.247) | (-2.317, 1.843) |  | (-3.286, 2.195) | (-3.757, 2.461) |
| Signs, symptoms and ill-defined | 5.066 | 2.255 |  | 2.185 | -1.549 |
|  | (-3.517, 13.65) | (-8.746, 13.26) |  | (-6.739, 11.11) | (-13.51, 10.41) |
| Homicide | 1.924 | -1.015 |  | -0.703 | -2.792 |
|  | (-3.723, 7.570) | (-8.283, 6.254) |  | (-5.387, 3.980) | (-8.282, 2.699) |
|  | **Infant** | |  |  |  |
| Infant mortality | -1.202 | -0.289 |  |  |  |
|  | (-2.633, 0.230) | (-1.534, 0.956) |  |  |  |

Notes:

a. Based on the available data from LIS, we constructed longitudinal data for 24 European countries with measurements for 1990/1995/2000/2005. If income inequality was not available for the year needed, Gini from the nearest year was used. If there were more than 1 neighboring year, the linear interpolation was used. The included countries were Austria, Belgium, Czech Republic, Denmark, Estonia, Finland, Germany, Greece, Hungary, Ireland, Italy, Luxembourg, Netherlands, Norway, Poland, Romania, Russia, Slovak Republic, Slovenia, Spain, Sweden, Switzerland and United Kingdom.

b. Model 1 includes Gini index (95% confidence interval in parentheses, based on clustered standard errors), year dummies, log(gdp) and country fixed effects.

c. Model 2 additionally adds democracy index, education, independence, armed conflict and economic freedom.
